# Mycobacterium fortuitum and Mycobacterium abscessus infections in the foot and ankle in two immunocompetent patients

**DOI:** 10.37796/2211-8039.1021

**Published:** 2020-12-01

**Authors:** Khai Phang Wong, Zhi Hao Tang, Gek Meng Tan

**Affiliations:** Department of Orthopaedic Surgery, Khoo Teck Puat Hospital, Singapore

**Keywords:** conventional chemotherapy, musculoskeletal infections, nontuberculous mycobacteria, osteomyelitis, septic arthritis

## Abstract

Nontuberculous mycobacteria (NTM) infections of the musculoskeletal system are commonly missed due to its rarity and the absence of systemic symptoms. A high clinical index of suspicion is required to recognize such infections as they may occur in immunocompetent hosts. We present two cases of foot and ankle NTM infections involving *Mycobacterium fortuitum* and *Mycobacterium abscessus* in two such patients. The first case involves an 83-year old lady who presented with a two-month history of multiple foot abscesses initially treated at a rural hospital. She underwent drainage and debridement of her foot, with eventual cultures growing *Pseudomonas aeruginosa* and *Mycobacterium abscessus*. She was initially treated with clarithromycin and doxycycline. At one year follow-up review, her wound healed completely. The second case involves a 55-year old man who presented with infection following midfoot fusion and anterolateral thigh flap for an open complex fracture dislocation of his right foot. Cultures eventually grew *Mycobacterium fortuitum* and he was treated with cefoxitin, clarithromycin and doxycycline. 10 months after his initial injury, the infection has cleared and his flap was clean. Through these 2 cases, we hope to highlight the unusual presentations of such infections and illustrate that with a high initial index of suspicion and appropriate treatment, these infections can be treated successfully.

## 1. Introduction

Nontuberculous mycobacteria (NTM) infections are defined as infections caused by *Mycobacterium* other than *Mycobacterium tuberculosis* and *Mycobacterium leprae*. They can be found in the environment in soil, water, vegetables, domestic animals and dairy products. There have been >120 recognized species of NTM, 60% of which have been known to cause human diseases [1]. Most NTM infections cause pulmonary diseases, while musculoskeletal NTM infections are very rare[2]. Timely diagnosis may be difficult due to difficulty in growing the organism in culture, and delayed diagnosis may result in joint destruction and functional impairment. We present two cases of nontuberculous mycobacteria infections in the ankle in two immunocompetent patients.

## 2. Case reports

### 2.1. Case 1

An 83-year-old Chinese lady, presented in November 2011 with a two-month history of multiple left foot abscesses. She had no other significant past medical history. There was no history of trauma. She was initially treated in a rural hospital in a neighbouring country in which she underwent multiple wound debridements. She was started on ciprofloxacin and subsequently switched to bactrim due to allergy. She subsequently presented to our hospital with increasing foot pain and discharge.

**Fig. 1:**
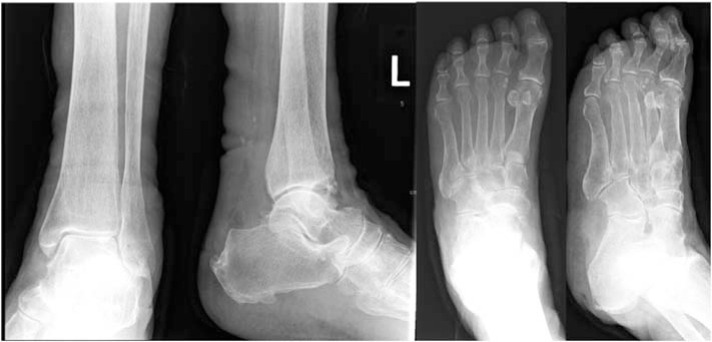
Radiographs of a patient who presented with multiple foot abscesses, showing osteopenic bones without erosive changes.

On examination, there were multiple sinuses on her left foot with purulent discharge. The range of motion of her left ankle was limited by pain. Distal pulses were palpable and sensation was intact. Radiographs revealed osteopenic bones but there were no erosive changes (Fig. 1). Her white cell count was 11.17×10^9^/L, C-reactive protein was 48.6 mg/L and erythrocyte sedimentation rate was 140 mm/hr. She was started on intravenous cefazolin.

The patient underwent left foot drainage and debridement. Intraoperatively, there were multiple draining sinuses and pus over the medial ankle and foot. The medial ankle sinus was found to be communicating with the ankle joint and an ankle arthrotomy was performed. There were multiple pockets of pus involving the ankle and subtalar joints, retrocalcaneal space and flexor hallucis longus tendon sheath. Tissue samples and pus were sent for cultures and histology.

Histological examination revealed active chronic inflammation with no evidence of neoplasia. Tissue cultures grew *Pseudomonas aeruginosa* and *Mycobacterium abscessus*, with sensitivities as shown in Table 1. After consultation with infectious disease specialists, clarithromycin 500md BD and doxycycline 100 mg BD was started. This was continued for 2 months. At 2 months follow-up, a bedside incision and drainage was done and tissue samples grew *Pseudomonas aeruginosa* and *Staphylococcus aureus*. She was switched to ciprofloxacin and cloxacillin for one more month. At one year follow-up review, her wound has healed completely.

### 2.2. Case 2

**Fig. 2:**
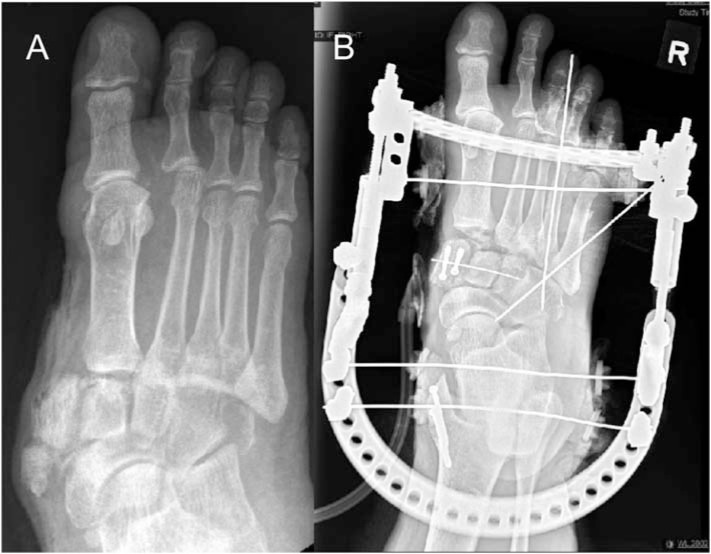
A: Radiograph showing right foot Lisfranc dislocation with cuneiform and cuboid fractures.B: Radiograph post-external fixation and midfoot arthrodesis.

A 55-year-old man with recently diagnosed diabetes was involved in a road traffic accident in March 2018. He was the driver of a mini-van which collided with a lorry. He sustained a right hip posterior dislocation and a right acetabular fracture for which he underwent fixation. He also sustained a right foot open lisfranc dislocation with fractures of all 3 cuneiforms and cuboid. He underwent external fixation for his right foot and subsequently midfoot arthrodesis (Fig. 2). Intraoperative cultures were negative and the open wound was eventually covered with an anterolateral thigh flap.

The patient subsequently had 3 readmissions for flap infection. The first admission occurred 4 months after injury. Initial wound swab grew methicillin-resistant *Staphylococcus aureus* (MRSA). During the wound debridement, there was a sinus over the flap with purulent discharge. The medial cuneiform screws were exposed and the medial cuneiform was unstable. The medial cuneiform was excised with removal of the exposed screws and a cement spacer was inserted. Intraoperative cultures were negative for bacterial growth. An infectious disease consult was made and the patient was started on intravenous vancomycin 1000 mg q12hrly for a total of 6 weeks duration.

The patient was readmitted for the second time 5 months after injury. Seropurulent discharge was noted during wound debridement and intraoperative cultures were again negative for bacterial growth. Decision was made to continue with intravenous vancomycin after consultation with infectious disease specialists.

The patient was readmitted 6 months after injury with new abscesses noted over his foot flap. He underwent debridement and seropurulent discharge was again noted in the wound. Intraoperative cultures grew *Mycobacterium fortuitum* (sensitivities as shown in Table 2). His antibiotics was switched to IV cefoxitin 2g q6hrly, PO clarithromycin 500 mg OM and PO doxycycline 100 mg BD for 6 weeks.

**Fig. 3:**
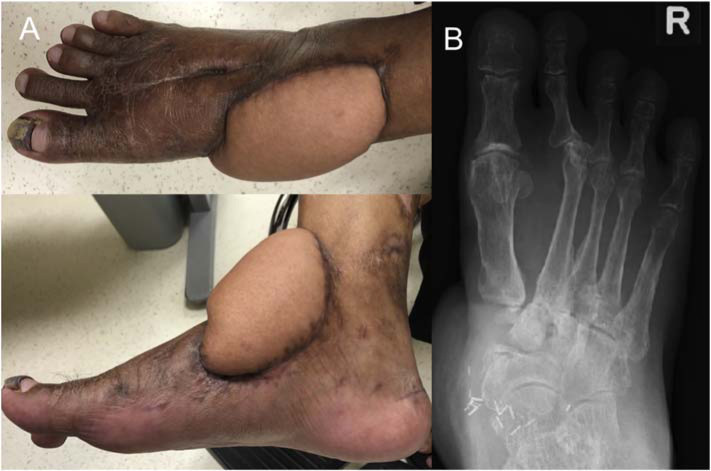
A: Clinical photos of the patient's flap 10 months post-injury. B: Radiograph at latest follow-up.

The patient is still on follow-up at the time of writing. During the latest outpatient visit 10 months post-injury, his flap was clean and healthy with no signs of infection (Fig. 3). He was also seen by the infectious disease specialist who recommended to continue PO clarithromycin and doxycycline for a total of 6 months.

## 3. Discussion

NTM infection is becoming an increasing problem worldwide. There have been >120 species of NTM reported, with about 60% identified as human pathogens[1]. They are important environmental pathogens that cause a broad spectrum of diseases in both immunocompetent and immunocompromised hosts. These diseases include infections of the pulmonary system, bone, eye, ear and central nervous system.

Musculoskeletal NTM infection has been found be generally associated with immunocompetent patients[3,13], with NTM infections in immunocompromised patients usually presenting with disseminated disease. Wagner and Young[13] found that rapidly growing mycobacteria (e.g. *M. marinum*, *M. avium*, *M. fortuitum*, *M. abscessus* and *M. chelonae*) were the most common NTM species found in immunocompetent individuals. On the other hand, some mycobacterial species (e.g. *M. chelonae*, *M. hemophilum*) are almost entirely recovered from immunocompromised individuals. A recent local study by Lim et al demonstrated that *M. abscessus* is the most common NTM isolated, with a higher prevalence in males and the elderly[2].

Most NTM infections cause pulmonary diseases, while musculoskeletal NTM infections are very rare [2]. Osteoarticular and soft tissue NTM infections are usually acquired through direct inoculation from environmental pathogens or via contiguous infection foci from surgical procedures or penetrating trauma[1]. The hand/wrist is reported to be the most common site of musculoskeletal NTM infections[1,3], with the foot and ankle being an extremely rare site of infection. There have been various case reports in the literature of ankle septic arthritis caused by various nontuberculous mycobacterial strains[4,5,6]. However, to our knowledge, no study has reported septic arthritis from *Mycobacterium abscessus*, a mycobacterium species more commonly associated with lung and skin/soft tissue infections. While there have been isolated reports of *Mycobacterium abscessus* in the foot presenting following penetrating injury or surgery[7,8,9], atraumatic presentation with multiple draining sinuses has never been described in the literature. As the patient did not have any history of trauma or penetrating injury, we postulate that soil exposure from a rural environment may be a possible cause of this infection. Similarly, while *Mycobacterium fortuitum* has been reported in various pulmonary, musculoskeletal, soft tissue and even prosthetic device infections[10,11], there has been no previous report on *Mycobacterium fortuitum* causing infection following foot implant surgery. By highlighting these two cases, we hope to create awareness of unusual presentations of nontuberculous mycobacteria and how they are treated.

Timely diagnosis may be difficult due to difficulty in growing the organism in culture. In our centre, all specimens obtained were stained by the Ziehl-Neelsen method according to the American Thoracic Society guidelines[12]. NTM species were identified by DNA reverse hybridization (INNOLiPA MYCOBACTERIA v2, Innogenetics NV, Ghent, Belgium) and high-performance liquid chromatography[2].

Piersimoni and Scarparo reported the average time between symptom onset and diagnosis of an NTM infection was approximately 10 months[1]. Our case reports demonstrate a shorter time to diagnosis with approximately 3 months for the first subject and 6 months in the second subject. For the second subject, we postulate that initial bacterial isolation of MRSA may mask or delay diagnosis of NTM. Initial AFB smears may be negative due to the time required for incubation of NTM in the laboratory.

This highlights the importance of a high clinical index of suspicion to prevent delayed diagnosis thereby resulting in joint destruction and functional impairment immunocompetent patients. Diagnosis must be considered in patients with increasing musculoskeletal system signs, those with inflammation after penetrating or blunt trauma or those with underlying risk factors undergoing a medical procedure[1]. Tissue cultures are mandatory for definitive diagnosis of the causative organism.

Although antimicrobial chemotherapy combined with surgical debridement is the recommended treatment for musculoskeletal infections, standardized treatment protocols for musculoskeletal NTM infections are currently unavailable. Traditionally, NTM infections are known to be difficult to treat and do not respond to conventional antituberculous agents[14]. Clarithryomycin is emerging as a drug of choice for localized disease caused by *M. fortuitum* and *M. abscessus*. *M. fortuitum* treatment usually consists of 2 orally administered agents, one of which is clarithromycin (500 mg b.d.), and the other either doxycycline (100 mg o.d.), trimethoprim-sulfamethoxazole, levofloxacin, moxifloxacin or gatifloxacin. The recommended duration of treatment is 4-6 months. *M. abscessus* treatment also involves a course of clarithromycin (500 mg b.d.) for 4-6 months[13]. More recent studies have shown that conventional chemotherapy and anti-mycobacterial agents are able to achieve similar good clinical outcomes[3,4]. In our series, both our patients responded well to conventional agents.

## 4. Conclusion

Our case reports demonstrate two unusual presentations of NTM infections in the foot and ankle treated with conventional antimicrobial agents with good outcome.

## Figures and Tables

**Table 1:** Antibiotic susceptibility profile to P. aeruginosa and M. abscessus.

Antibiotic	Susceptibility profile	MIC (mg/L)
**Pseudomonas aeruginosa**		
Ceftazidime	S	
Piperacillin/tazobactam	S	
Gentamicin	S	
Ciprofloxacin	S	
**Mycobacterium abscessus**		
Cefoxitin	I	64
Amikacin	I	32
Ciprofloxacin	R	>4
Clarithromycin	S	0.12
Doxycycline	R	>16
Linezolid	I	16

MIC: minimum inhibitory concentration, S: sensitive, I: intermediate, R: resistant

**Table 2:** Antibiotic susceptibility profile to M. fortuitum.

Antibiotic	Susceptibility profile	MIC (mg/L)
**Mycobacterium fortuitum**		
Moxifloxacin	S	<0.25
Cefoxitin	I	32
Imipenem	I	8
Amikacin	S	<1
Ciprofloxacin	S	1
Clarithroymycin	S	1
Doxycycline	S	0.25
Linezolid	R	32

MIC: minimum inhibitory concentration, S: sensitive, I: intermediate, R: resistant.
